# Supplementary material for: Phage predation accelerates the spread of plasmid-encoded antibiotic resistance
Source: Nat Commun. 2024 Jun 26;15:5397. doi: 10.1038/s41467-024-49840-7 (PMC11208555; doi:10.1038/s41467-024-49840-7)
Supplement: Supplementary file 1 — Supplementary Information [file 41467_2024_49840_MOESM1_ESM.pdf]

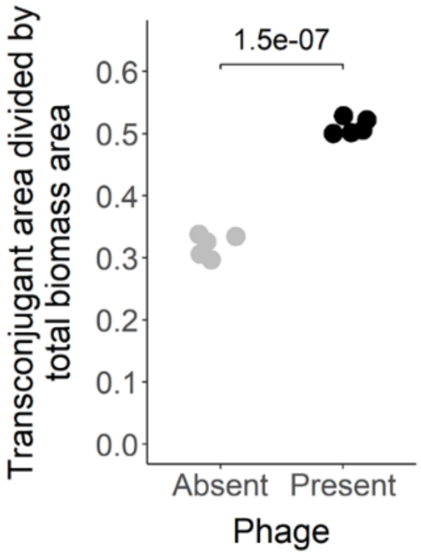

Supplementary Fig. 1: Total transconjugant area divided by the total biomass area at a fixed radial distance from the biomass centroid. We calculated the number of transconjugants using the same images that we used for Fig. 2 in the main text. We selected a fixed radial distance of 2100 μm, which is the minimum distance that is present across all of our samples. Each datapoint is a measurement for an independent experimental replicate (n = 5) and the *P* value is for a two-sample two-sided Welch test. Source data are provided as a Source Data file.

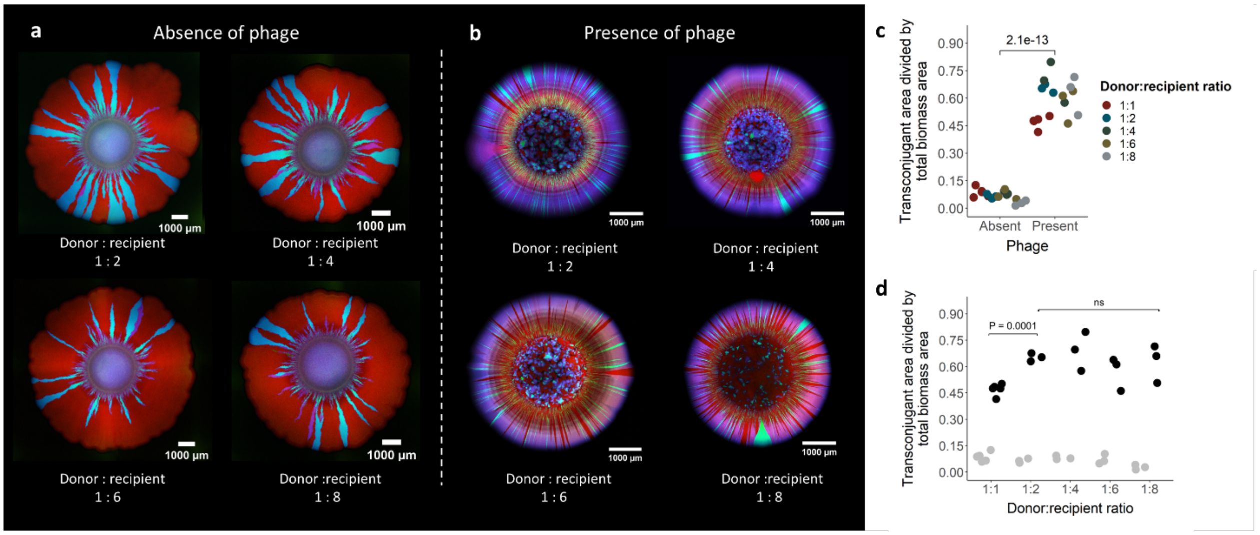

Supplementary Fig. 2: Surface-associated growth experiments with different initial ratios of the R388 donor to potential recipient. a,**b**, Representative CLSM images (n = 3) of the R388 donor and potential recipient after ten days of growth in anoxic conditions in the (**a**) absence or (**b**) presence of phage, where we added the phage directly to the growing biomass. The R388 donor expresses GFP and CFP and appears cyan, the potential recipient expresses RFP and appears red, and transconjugants express RFP and CFP and appear magenta. **c**, The total transconjugant area divided by the total biomass area for different initial ratios of the R388 donor to potential recipient. **d**, The total transconjugant area divided by the total biomass area plotted as a function of the initial ratio of the R388 donor to the potential recipient. For **c** and **d**, each datapoint is a measurement for an independent experimental replicate (n = 3) and the *P* values are for two-sample two-sided Welch tests. Source data are provided as a Source Data file.

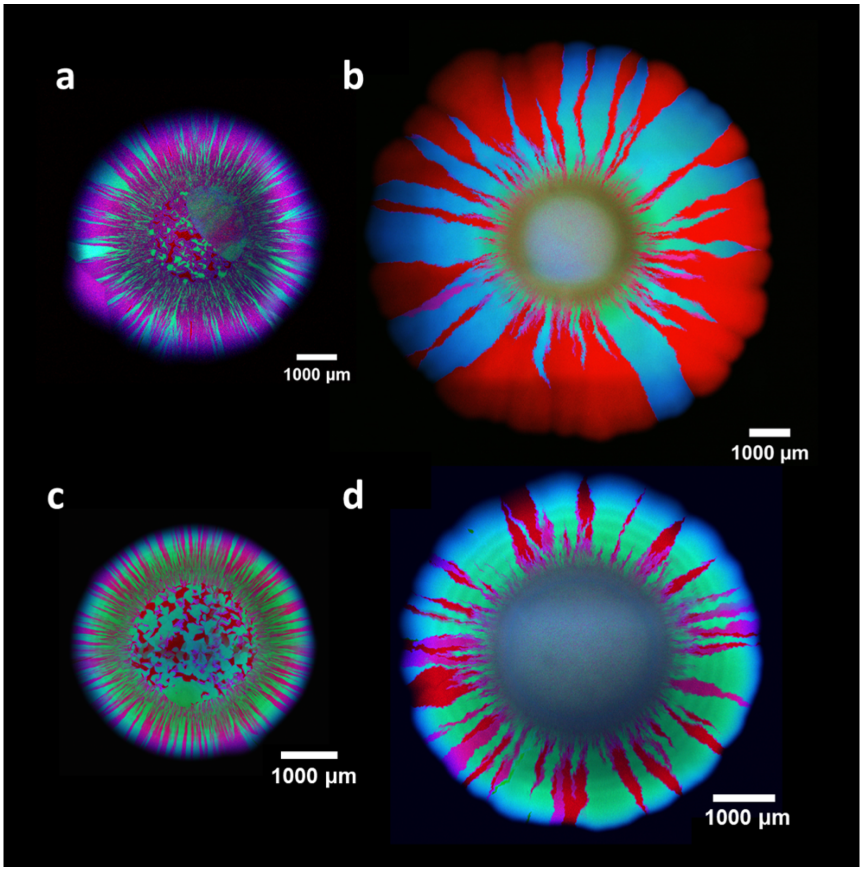

Supplementary Fig. 3: Surface-associated growth experiments in oxic and low nutrient conditions. **a**,**b**, Representative CLSM images (n = 5) of the R388 donor and potential recipient after six days of growth in the (**a**) presence or (**b**) absence of phage in oxic conditions, where we added the phage directly to the growing biomass. **c**,**d**, Representative CLSM images (n = 5) of the R388 donor and potential recipient in the (**c**) presence or (**d**) absence of phage after six days of growth on 10% LB agar plates, where we added the phage directly to the growing biomass. The R388 donor expresses GFP and CFP and appears cyan, the potential recipient expresses RFP and appears red, and transconjugants express RFP and CFP and appear magenta recipient expresses RFP and appears red, and transconjugants express RFP and CFP and appear magenta.

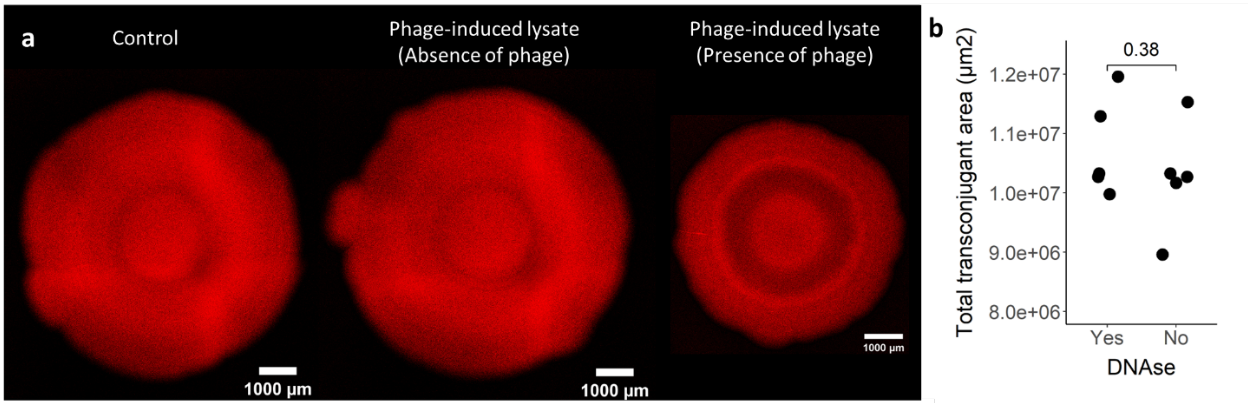

Supplementary Fig. 4: Experimental tests of whether R388 or its associated genes are transferred via natural transformation or transduction. **a**, Representative CLSM images (n = 5) of the potential recipient grown alone for ten days in anoxic conditions. Control; the potential recipient grown without phage or phage-induced lysate. Phage-induced lysate (Absence of phage); the potential recipient grown with phage-induced lysate of the R388 donor and with heat-inactivated phage. Phage-induced lysate (Presence of phage); the potential recipient grown with phage-induced lysate of the R388 donor and with viable phage. **b**, Total number of transconjugants that emerge with or without DNase I digestion of free DNA. Each datapoint is a measurement for an independent experimental replicate (n = 5) and the *P* value is for a two-sample two-sided Welch test. Source data are provided as a Source Data file.

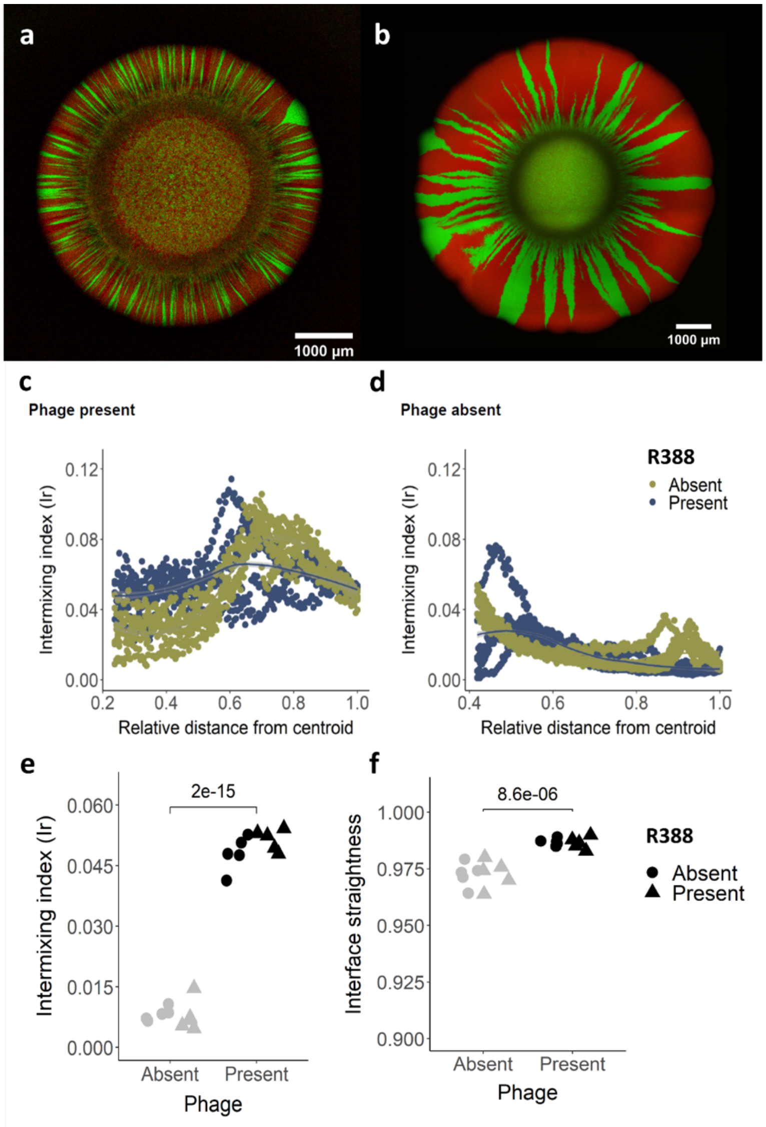

Supplementary Fig. 5: Effect of phage on spatial intermixing and interface straightness in the absence or presence of R388. a,**b**, Representative CLSM images (n = 5) of two *E. coli* strains lacking R388 after ten days of growth in anoxic condition in the (**a**) presence or (**b**) absence of phage, where we added the phage directly to the growing biomass. **c**,**d**, The intermixing index as a function of the radial distance from the centroid of the inoculation area to the final biomass periphery in the (**c**) presence or (**d**) absence of phage. Source data are provided as a Source Data file. **e**, The intermixing index quantified across a 30 µm thick band positioned at the final biomass periphery. **f**, The mean interface straightness. For **e** and **f**, each data point is a measurement for an independent experimental replicate (n = 5) and the *P* values are for two-sample two-sided Welch tests. Source data are provided as a Source Data file.

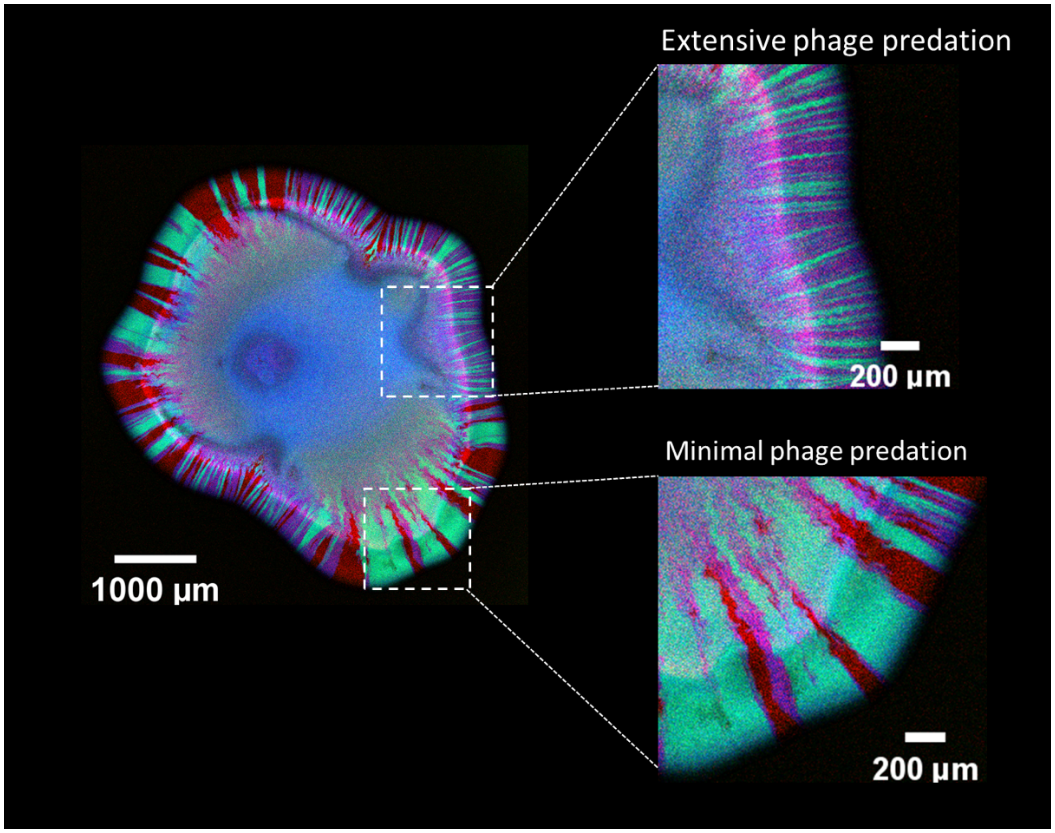

Supplementary Fig. 6: Surface-associated growth experiments with the direct amendment of a low dosage of phage. Representative CLSM image (n = 5) of the R388 donor and potential recipient after ten days of growth in anoxic conditions where we added the phage directly to the growing biomass at a low dosage of 6 × 10^4 PFU/mL. The R388 donor expresses GFP and CFP and appears cyan, the potential recipient expresses RFP and appears red, and transconjugants express RFP and CFP and appear magenta. The images on the right are magnifications of the regions in the white dashed boxes in the image on the left. The upper right box is a representative region with extensive phage predation and extensive R388 transfer while the lower right box is a representative region with minimal phage predation and minimal R388 transfer.

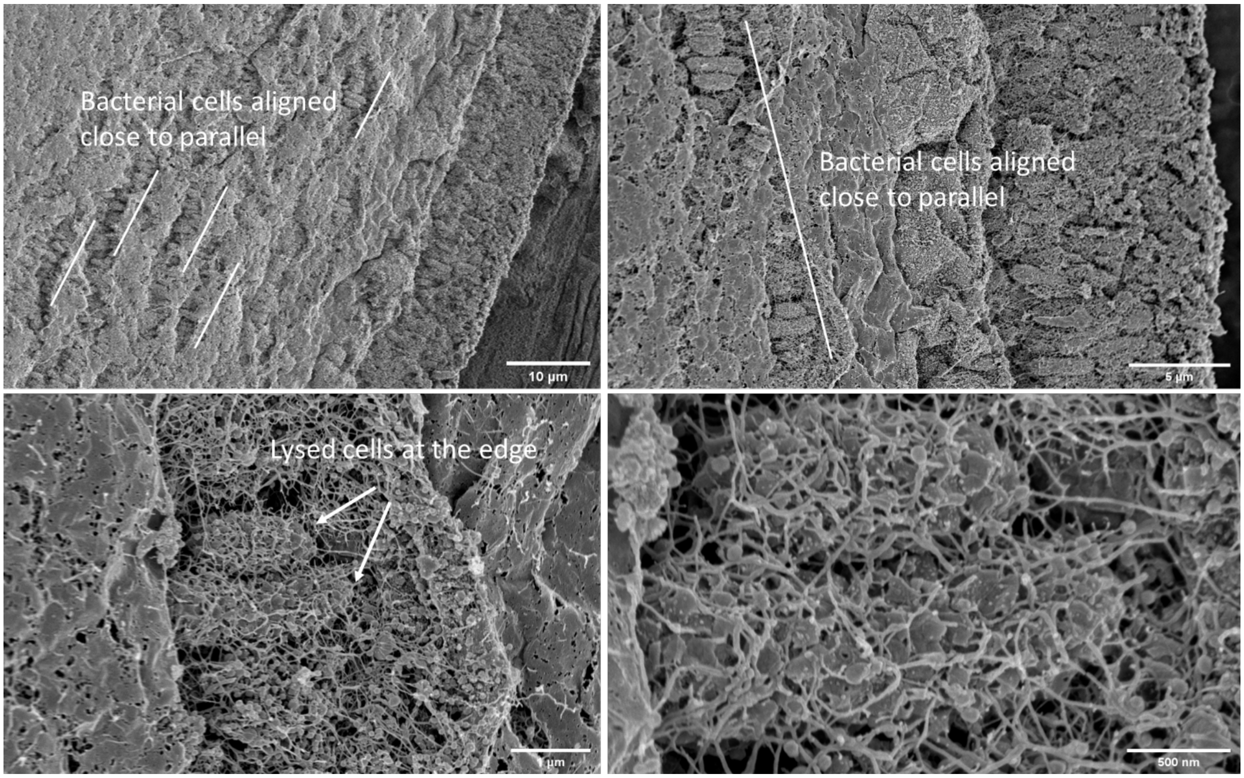

Supplementary Fig. 7: Representative scanning electron microscopy images (n = 5) of surface-associated growth in the presence of phage. The experimental setup is identical to that used for the data presented in Fig. 2. Briefly, we inoculate the R388 donor and potential recipient onto the center of a nutrient-amended agar surface. After the bacteria to grow for six hours, we deposit a 1 µl droplet of the phage solution directly on the growing biomass. After ten days of incubation in anoxic conditions, we then analyze the biomass with scanning electron microscopy. During phage predation, the cells are aligned close to parallel with each other.

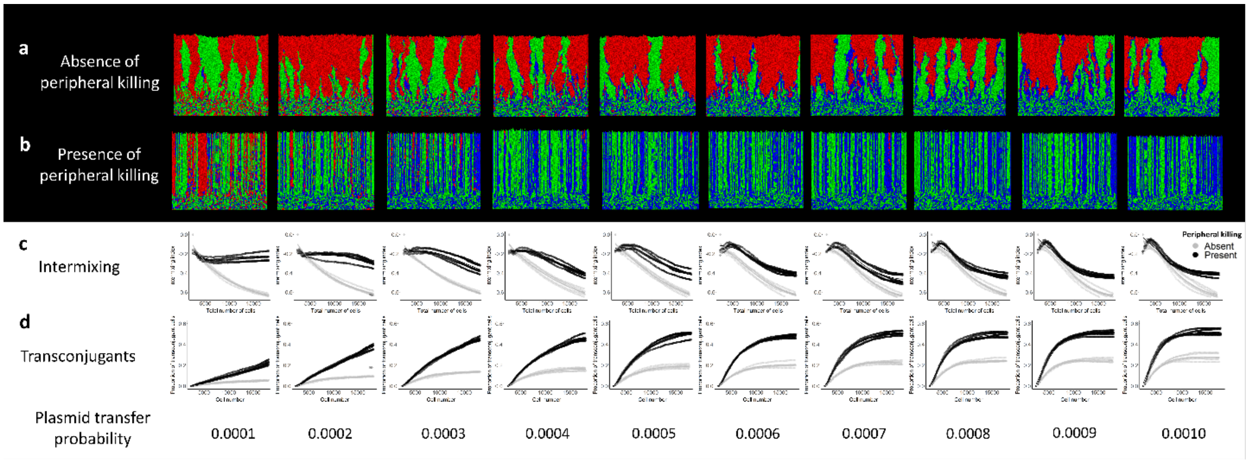

Supplementary Fig. 8: Surface-associated growth simulations in the presence of a plasmid as a function of the plasmid transfer probability. a,**b**, Representative simulations (n = 5) of two competing strains where the red cells are potential recipients and the green cells carry a plasmid that reduces the growth rate by 5%. If a red cell receives the plasmid from a green cell, its growth rate is reduced accordingly and appears blue. We simulated biomass growth as a function of the plasmid transfer probability in the (**a**) absence or (**b**) presence of peripheral killing until reaching a population size of 18,000 cells. **c**, The intermixing index as a function of the radial distance from the centroid of the inoculation area to the final biomass periphery. **d**, The frequency of transconjugants as a function of the radial distance from the centroid of the inoculation area to the final biomass periphery. For **c** and **d**, data are plotted in the presence (black lines) or absence (grey lines) of peripheral killing. Each line is for an independent simulation (n = 5).

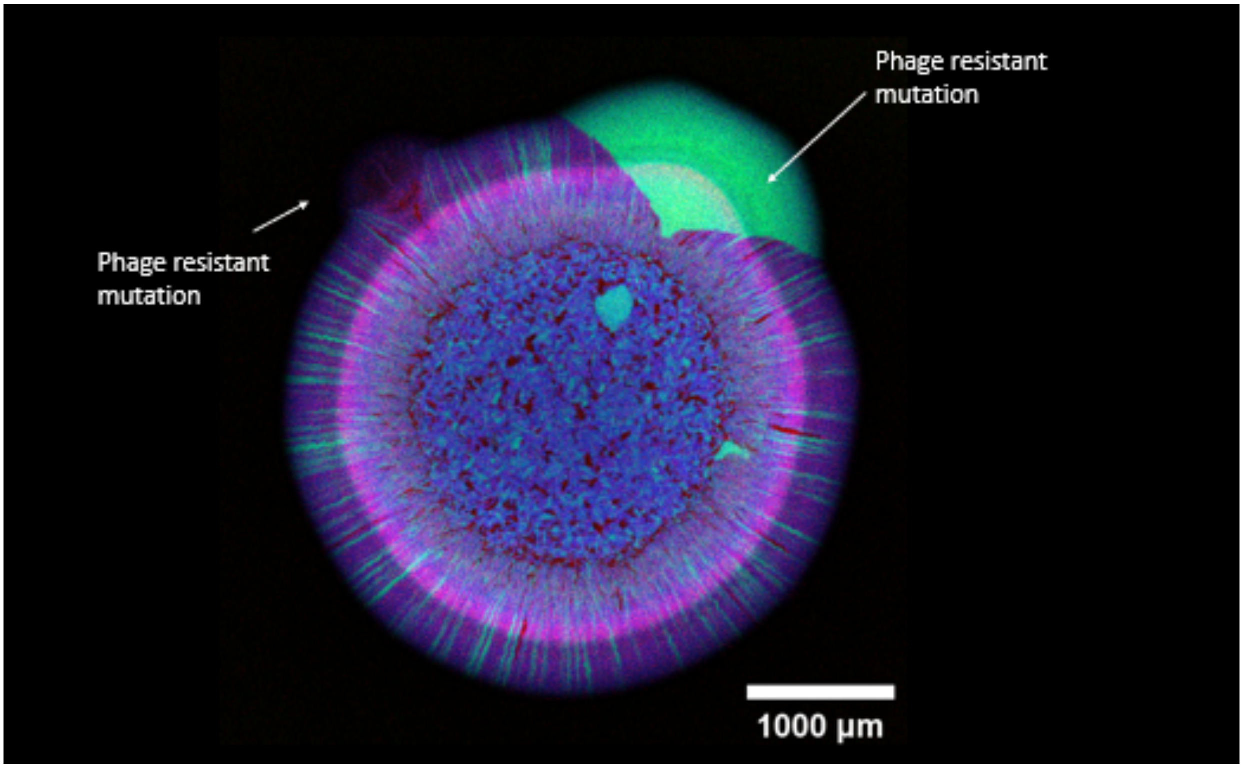

Supplementary Fig. 9: Representative CLSM image (n = 5) of a phage-resistant mutant emerging during surface-associated growth. The R388 donor and potential recipient after six days of growth in anoxic conditions in the presence of phage, where we directly added the phage to the growing biomass. The R388 donor expresses GFP and CFP and appears cyan, the potential recipient expresses RFP and appears red, and transconjugants express RFP and CFP and appear magenta.

Parameter	Description	Value	Unit
g_n	Specific growth rate when not carrying the plasmid	1	-
$g_{plasmid}$	Specific growth rate when carrying the plasmid	0.95	-
L_0	Mean initial cell length	2	µm
L_d	Cell length at division	3.5-4	µm
$w_{infection}$	Width of the peripheral cell layer susceptible to killing	4	µm
d_{cell}	Mean diameter of the cells	0.5	µm
N	Initial cell number	1200	cell
P	Probability of horizontal gene transfer	0.0001-0.001	-

Supplementary Table 1: Model parameters used for individual-based computational simulations of surface-associated microbial growth in the presence of a plasmid.
