# Supplementary material for: Phage predation accelerates the spread of plasmid-encoded antibiotic resistance
Source: Nat Commun. 2024 Jun 26;15:5397. doi: 10.1038/s41467-024-49840-7 (PMC11208555; doi:10.1038/s41467-024-49840-7)
Supplement: Supplementary file 3 — Description of Additional Supplementary Files [file 41467_2024_49840_MOESM3_ESM.pdf]

File Name: Supplementary Video 1

Description: Simulations of spatial self-organization during surface-associated growth. We simulated three conditions; in the presence of peripheral killing, in the absence of peripheral killing, and in the absence of peripheral killing but where the cell growth rate is set to zero for cells located in the outer two layers at the biomass periphery (interior growth). The initial number of green and red cells is 600 each, where the green and red cells are phenotypically identical except having different colors. We performed simulations until reaching 18,000 cells.

File Name: Supplementary Video 2

Description: Simulations of plasmid transfer during surface-associated growth with different plasmid transfer probabilities in the absence of peripheral killing. The initial number of green and red cells is 600 each, where the green cells are plasmid donors, the red cells are potential recipients, and the blue cells are transconjugants. We performed simulations for ten different plasmid transfer probabilities ranging from 0.0001 to 0.001 at increments of 0.0001. We performed simulations until reaching 18,000 cells.

File Name: Supplementary Video 3

Description: Simulations of plasmid transfer during surface-associated growth with different plasmid transfer probabilities in the presence of peripheral killing. The initial number of green and red cells is 600 each, where the green cells are plasmid donors, the red cells are potential recipients, and the blue cells are transconjugants. We performed simulations for ten different plasmid transfer probabilities ranging from 0.0001 to 0.001 at increments of 0.0001. We performed simulations until reaching 18,000 cells.
